# Influence of argon plasma on the deposition of Al_2_O_3_ film onto the PET surfaces by atomic layer deposition

**DOI:** 10.1186/1556-276X-8-79

**Published:** 2013-02-15

**Authors:** Riyanto Edy, Xiaojiang Huang, Ying Guo, Jing Zhang, Jianjun Shi

**Affiliations:** 1 College of Science, Donghua University, Shanghai, 201620, People’s Republic of China; 2 State Key Laboratory for Modification of Chemical Fibers and Polymer Materials, College of Material Science and Engineering, Donghua University, Shanghai, 201620, People’s Republic of China; 3 Member of Magnetic Confinement Fusion Research Center, Ministry of Education of the People’s Republic of China, Shanghai, 201620, People’s Republic of China

**Keywords:** Atomic layer deposition, PET film, Al_2_O_3_ coating

## Abstract

In this paper, polyethyleneterephthalate (PET) films with and without plasma pretreatment were modified by atomic layer deposition (ALD) and plasma-assisted atomic layer deposition (PA-ALD). It demonstrates that the Al_2_O_3_ films are successfully deposited onto the surface of PET films. The cracks formed on the deposited Al_2_O_3_ films in the ALD, plasma pretreated ALD, and PA-ALD were attributed to the energetic ion bombardment in plasmas. The surface wettability in terms of water contact angle shows that the deposited Al_2_O_3_ layer can enhance the wetting property of modified PET surface. Further characterizations of the Al_2_O_3_ films suggest that the elevated density of hydroxyl -OH group improve the initial growth of ALD deposition. Chemical composition of the Al_2_O_3_-coated PET film was characterized by X-ray photoelectron spectroscopy, which shows that the content of C 1*s* reduces with the growing of O 1*s* in the Al_2_O_3_-coated PET films, and the introduction of plasma in the ALD process helps the normal growth of Al_2_O_3_ on PET in PA-ALD.

## Background

Atomic layer deposition (ALD) is an ultrathin film deposition method by sequential exposure of gas phase reactants for the deposition of thin films with atomic layer accuracy [1-3]. Each atomic layer formed in the sequential process is a result of saturated surface controlled chemical reactions [4-6]. In plasma-assisted atomic layer deposition (PA-ALD), additional energy for the chemical reaction is provided by applying plasmas at an appropriate time interval during the reaction cycle, in which the plasmas are used to produce radicals by gas dissociation [4,7,8]. It brings the advantages of improving the reaction rates, the process efficiency, the fragmentation of precursor molecules, and the removal of product molecules [4,9]. The reactive surface groups play an important role for the initial growth and nucleation of Al_2_O_3_ thin film in atomic layer deposition by reacting with the precursor molecules [10-13]. Hydroxyl groups are considered to be the typical reactive groups, which secure a good adhesion of chemical bonding between the underlying substrate and the deposited thin film [5,13]. The ALD growth of Al_2_O_3_ on several polymers with and without reactive surface groups have been investigated with the precursors trimethylaluminum (TMA) and H_2_O [1,11,13], which demonstrated that the initial nucleation on polymers without the reactive surface groups were started by the strong absorption and retention of TMA in the surface layers of polymer, which reacted with the water vapor during the subsequent deposition pulse [11-13]. It suggests that the quality of deposited film depends on the surface coverage in the adsorption step, which is governed by the concentration and spatial distribution of reactive groups on the substrate [5,14]. It takes 10 to 20 ALD cycles to form the Al_2_O_3_ film on the polymer surface before the deposition achieves a normal ALD growth with the deposition rate similar to that observed in the other surfaces [13]. Unfortunately, the understanding of deposition dynamics in ALD by introducing the plasmas is incomplete. Here, studies on ALD and PA-ALD deposition on PET films with and without plasma pretreatment are carried out to demonstrate the influence of argon plasmas on the deposition of Al_2_O_3_ film.

## Methods

Polyethylene terephthalate (PET) film and silicon were used as the substrates. PET is a semi-crystalline polymer at room temperature, which is cleaned by an ultrasonic machine for 20 min with ultrasonic power and temperature of 80 W and 30°C, respectively. The films were dried in a vacuum oven for 1 h with temperature of 50°C.

**Figure 1:**
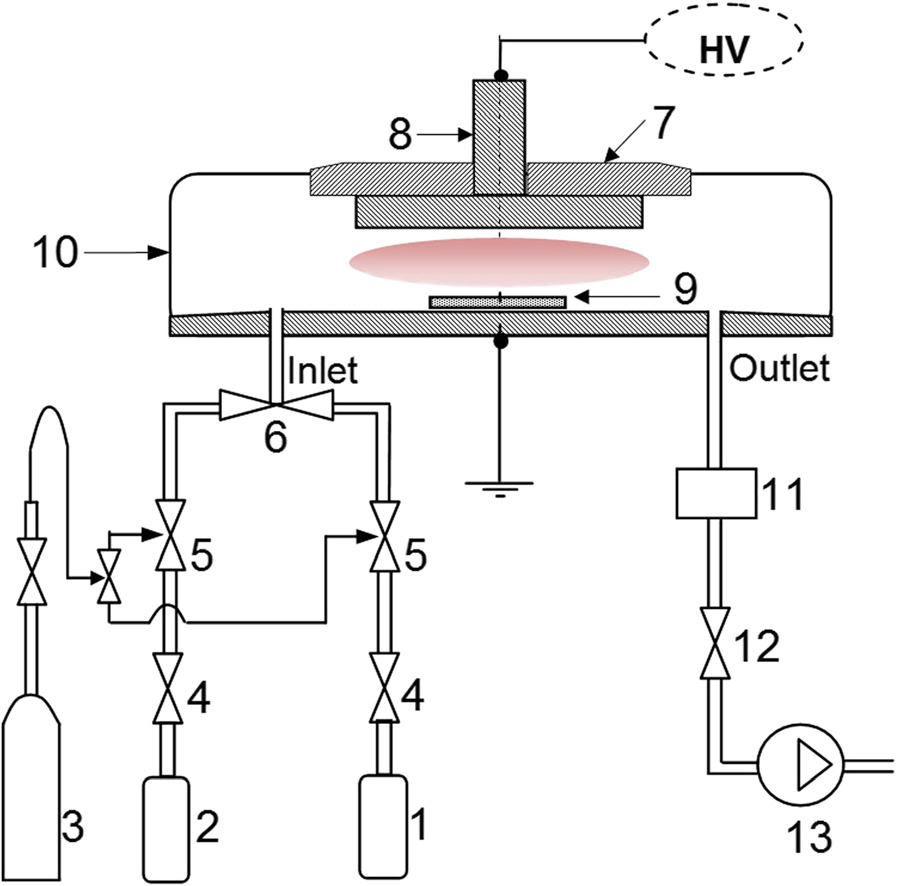
**Schematic of the PA-ALD process.** (1) H_2_O, (2) TMA, (3) Ar gas cylinder, (4) precursor control valve, (5) Ar control valve, (6) check valve, (7) isolator, (8) electrode, (9) substrate, (10) reactor, (11) pressure gauge, (12) needle valve, and (13) vacuum pump.

Aluminum oxide depositions onto the substrate were conducted by ALD and PA-ALD, whose schematic is shown in Figure 1. The precursors of trimethylaluminum (TMA/Al(CH_3_)_3_) and water vapor were sequentially exposed for 10 ms and purged for 10 s, respectively. The deposition temperature and deposition cycle were fixed at 90°C and 100. The plasma was ignited between two parallel stainless steel electrodes with the interelectrode distance of 10 mm by a radiofrequency power supply at 13.56 MHz and 20 W. The plasma pretreatment was conducted for 90 s. The pressure of the deposition processes within the reactor of ALD and PA-ALD was 24.43 and 36.1 Pa, respectively. The argon gas was functionalized as both the carrier gas and discharge gas with the flow rate of 20 sccm.

Cross section of the coated silicon and the front view of the coated PET film were imaged by field emission scanning electron microscopy (FESEM; Hitachi, S-4800, Tokyo, Japan). Contact angle measurement was conducted by the sessile drop technique on the surface of the PET films. Deionized water drop tests were carried out on each of the samples using 0.4-μl-size droplet on each testing. The wetting property level of Al_2_O_3_-coated PET film was measured by a static contact angle analysis system (JC2000A, Powereach, Shanghai, China). Atomic force microscopy (AFM; NanoScope IV SPM, Veeco, Plainview, NY, USA) was used to examine the surface morphology of the PET film before and after Al_2_O_3_ deposition using the tapping mode. Fourier transform infrared (FTIR) spectra of the films were measured using the Thermo Nicolet Nexus 670 (Thermo Scientific, Waltham, MA, USA), Smart iTR (diamond ATR, Thermo Scientific) with the spectral range of 650 to 4,000 cm^−1^ with the resolution of 4 cm^−1^. Chemical composition of Al_2_O_3_-coated PET film was evaluated by X-ray photoelectron spectroscopy (XPS).

## Results and discussion

### Surface morphology of the deposited Al_2_O_3_ film

**Figure 2:**
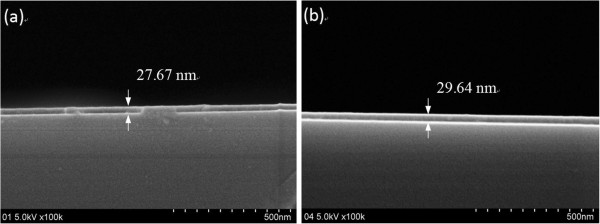
**Cross-sectional FESEM images of the aluminum oxide-coated silicon.** By **(a)** ALD and **(b)** PA-ALD.

Cross-sectional images of the aluminum oxide film deposited on the silicon substrate by ALD and PA-ALD are presented in Figure 2a,b, respectively. The FESEM images show that the deposited aluminum oxide films have a smooth surface with a thickness of approximately 27.67 and 29.64 nm by ALD and PA-ALD, respectively. It indicates that the aluminum oxide film can be deposited on the PET film in the same ALD reactor.

**Figure 3:**
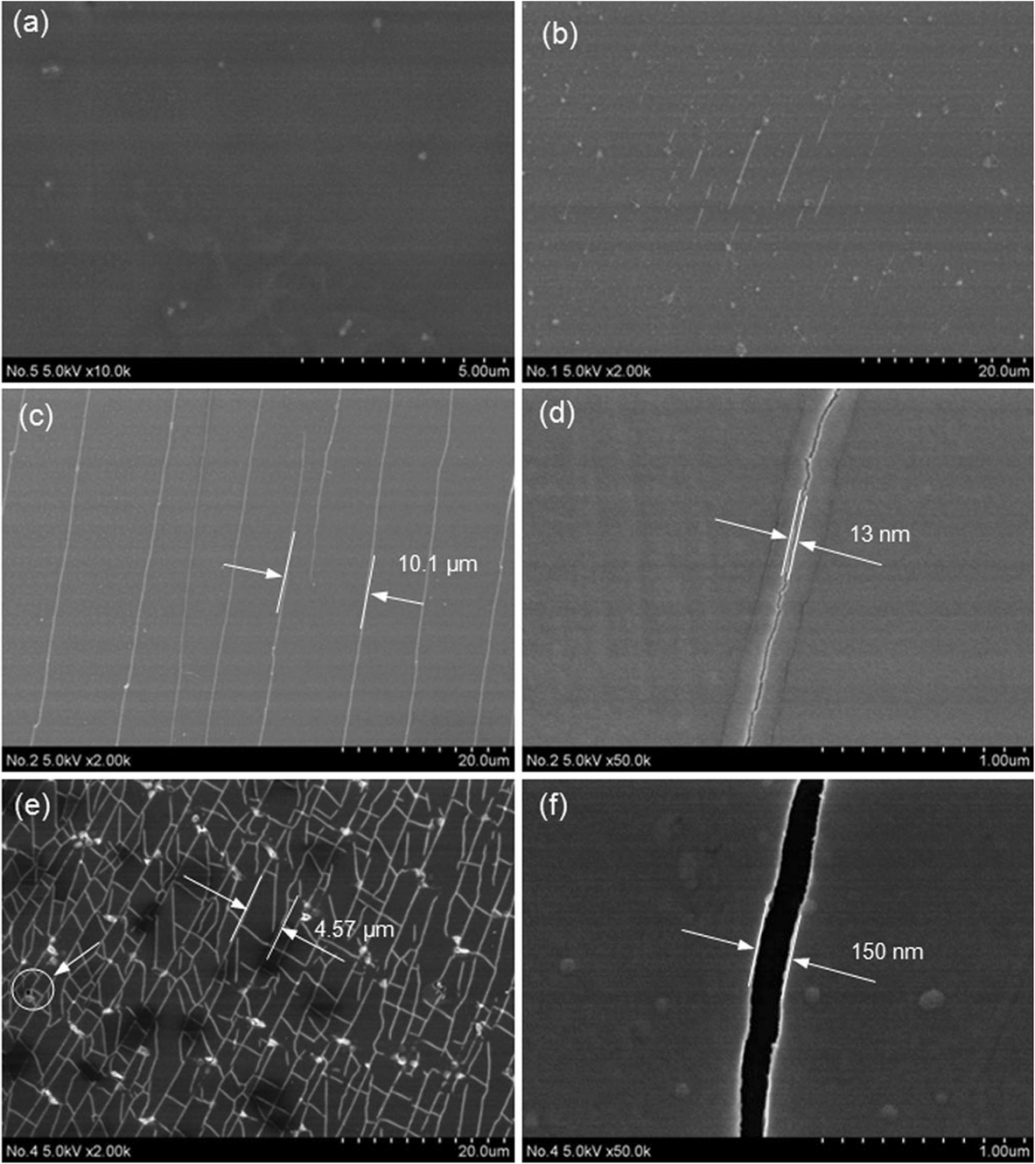
**FESEM images. (a)** Uncoated PET film and aluminum oxide-coated PET film by **(b)** ALD, **(c)** ALD with plasma pretreatment, and **(e)** PA-ALD. **(d)** and **(f)** are the magnified images of **(c)** and **(e)**.

Figure 3 shows the FESEM images of uncoated (Figure 3a) and aluminum oxide-coated PET films (Figure 3b,c,d,e,f). It shows cracks on the deposited Al_2_O_3_ films in ALD (Figure 3b), ALD with plasma pretreatment (Figure 3c), and PA-ALD (Figure 3d). The characteristics of the cracks in terms of density and gap distance are both enhanced by introducing the plasmas in ALD. The cracks show the same direction on the aluminum oxide films deposited by ALD and plasma pretreated ALD, as shown in Figure 3b,c. On the other hand, the cracks are intersectional on the aluminum oxide films deposited by PA-ALD, as shown in Figure 3e. The gap distance also increased from 13 to 150 nm for the cracks deposited by plasma pretreated ALD and PA-ALD, as shown in the magnified images of Figure 3d,f. The formation of cracks on the PET films is attributed to the crystallization of PET under the deposition temperature and the compressive stress induced by handling for the examinations [12], and most importantly, the introduction of plasmas in the ALD process [15].

It was shown that the cracks form above the aluminum oxide deposited on the PET films by plasma pretreated ALD and PA-ALD, during which the plasmas are responsible for not only the fragmentation of molecule precursors but also the detrimental effect on the aluminum oxide layers deposited on the PET surfaces. The energetic ion bombardment in the plasmas can create surface defect sites, which is considered to be the reason for the formation of cracks [15]. On the other hand, the energetic ion bombardment reduces the activation energy for chemisorption and limits the formation of solid compound [15], which fills the requirement for the self-limiting deposition in ALD wherein the binding energy of a monolayer chemisorbed on the surface is higher than the binding energy of subsequent layers on top of the formed layer.

Furthermore, the uppermost polymer surface with the Ar plasma exposure may incorporate the oxygen by cross-linking reactions. It induces the presence of trace oxygen to react with the precursor molecules that lead to the occurrence of numerous peel-off sites [16]. Although the cracks appear on the PET surface coated by ALD with plasma pretreatment and PA-ALD, the deposited surface area achieves the smooth state. It indicates that the necessary chemical functional groups induced due to the energetic ion bombardment in plasmas have a significant role on the initial growth on the PET surfaces.

**Figure 4:**
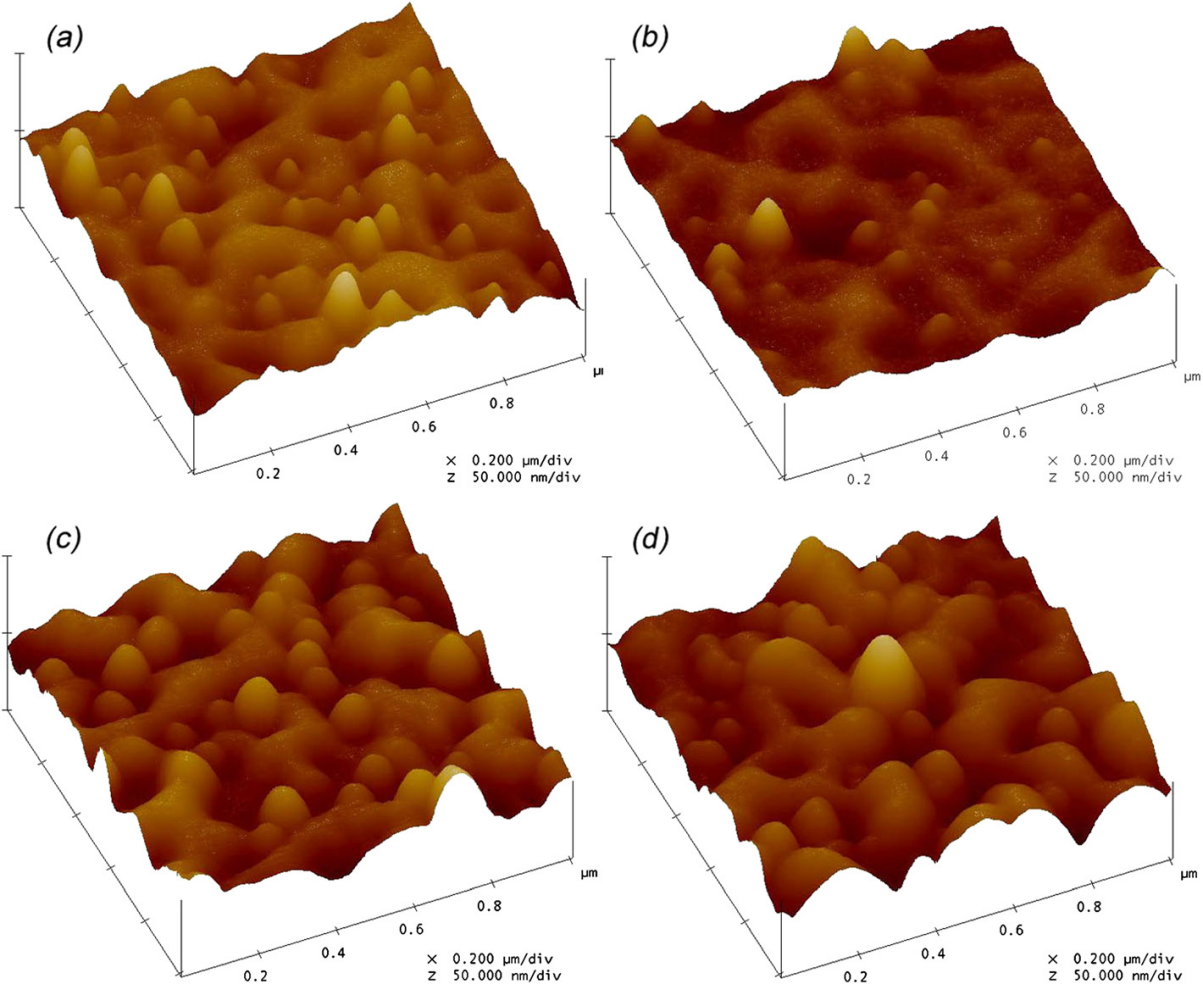
**AFM images. (a)** Uncoated PET film, the Al_2_O_3_-coated PET films by **(b)** ALD, **(c)** ALD with plasma pretreatment, and **(d)** PA-ALD.

The surface morphologies of Al_2_O_3_-coated PET films are shown in Figure 4. The root mean square (RMS) surface roughness is evaluated to be 7.9 and 7.2 nm for the uncoated PET film and the Al_2_O_3_ deposited PET film by ALD, respectively. With the introduction of plasmas in ALD, the RMS surface roughness is raised to be 8.1 and 9.8 nm for the Al_2_O_3_ deposited PET film by plasma pretreated ALD and PA-ALD, respectively. Given that the plasma provides the additional energy for chemical reactions in ALD process, the deposition of Al_2_O_3_ can be enhanced with the assistance of plasma in ALD.

### Wettability of the deposited Al_2_O_3_ film

**Figure 5:**
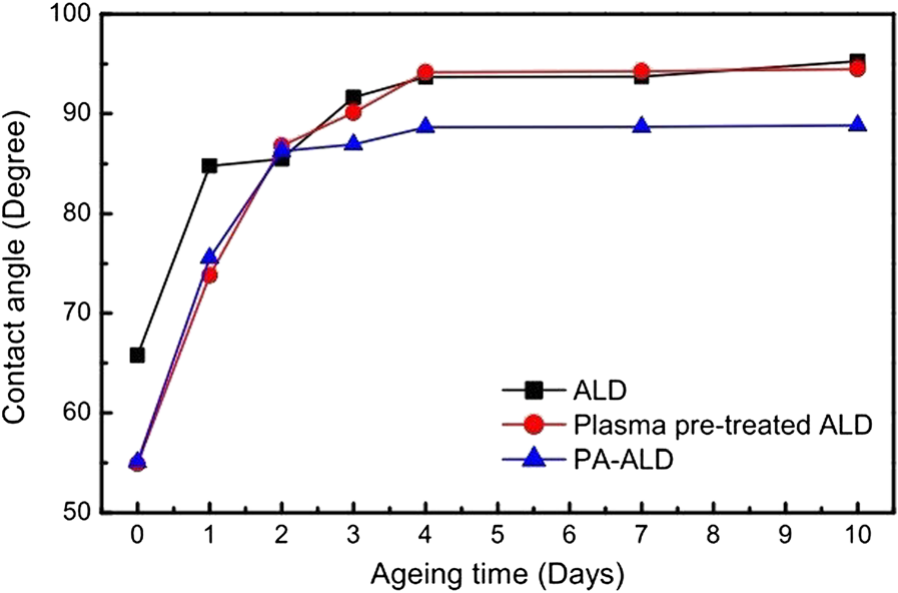
The water contact angle as a function of the aging time.

The wettability of the Al_2_O_3_ film on PET is examined by means of the water contact angle measurement, as shown in Figure 5. It clearly demonstrates the significant improvement of wettability when the water contact angle reduces to 65.76° with the deposition of Al_2_O_3_ film on PET by ALD, compared to the contact angle of the uncoated substrate (88.26°). The enhancement of wettability is attributed to the surface rearrangement by the ALD coating of aluminum oxide. Further reduction of contact angle is achieved to be 54.9° and 55.07° by the plasma pretreated ALD and PA-ALD, respectively, which suggests that the introduction of plasma in ALD provides additional ion bombardment on the deposited Al_2_O_3_ film. It proposes that the plasma employed in ALD contributes to both the fragmentation of precursor molecules and the surface activation of PET surfaces.

Figure 5 also shows the recovering of water contact angle as a function of time. It shows that the induced modifications on the wettability of the Al_2_O_3_ film on PET are not permanent since the contact angle increases to around 86° in about 2 days, which approaches that of the uncoated PET film. The recovering of water contact angle suggests the decrease of surface free energy with aging time [17], which is caused by the reorientation of induced polar chemical groups into the bulk of the material [18,19]. It is also worth noting that the water contact angles of Al_2_O_3_ films deposited by ALD and plasma pretreated ALD (approximately 94°) are higher than that of PA-ALD (approximately 88°) after 3 days of aging. It can be attributed to the cracks formed on the deposited Al_2_O_3_ film, as shown in Figure 3, which also reduce the water contact angle.

**Figure 6:**
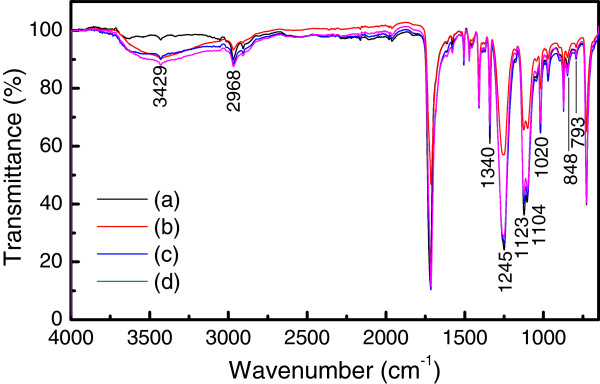
**FTIR spectra. (a)** Uncoated PET, the Al_2_O_3_-coated PET films by **(b)** ALD, **(c)** ALD with plasma pretreatment, and **(d)** PA-ALD.

The surface modification by Al_2_O_3_ deposition is considered to be mostly responsible for the reduction of water contact angle, although the cracks on the deposited Al_2_O_3_ film also contributes to the reduction of water contact angle, which is confirmed by the FTIR measurements, as shown in Figure 6. The changes in the FTIR spectra are clearly found at the bands of 793, 848, 1,020, 1,123 to 1,104, 1,245, 1,340, 3,429, and 2,968 cm^−1^, [20-23]. Among them, the absorption peak at 3,429 cm^−1^, corresponding to the hydroxyl group (−OH) [20,23], plays an important role in the film growth in ALD and the reduction of water contact angle.

The amplitude of the absorption peak at 3,429 cm^−1^ is found to be enhanced with the Al_2_O_3_ deposition by ALD, especially with the introduction of plasmas in ALD, which suggests the elevated density of -OH group on the surface of Al_2_O_3_ film deposited by PA-ALD. The -OH groups, acting as the reactive nucleation sites, are important to improve the quality of the deposited films in terms of uniformity and conformal film coverage without substantial subsurface growth [24].

### Chemical composition of the deposited Al_2_O_3_ film

**Figure 7:**
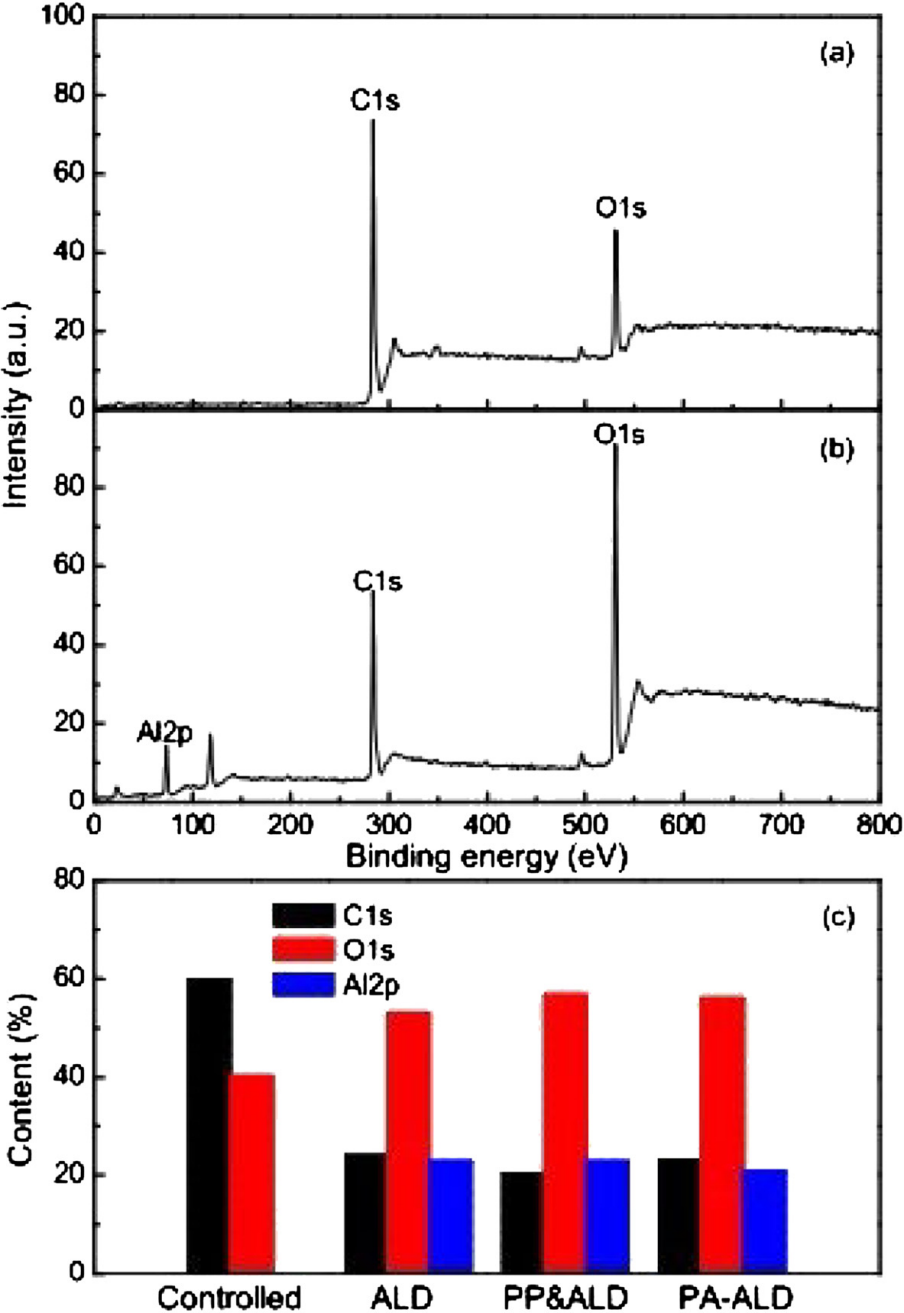
**XPS spectra. (a)** Uncoated PET, **(b)** the Al_2_O_3_-coated PET film by PA-ALD, and **(c)** relative elemental contents.

**Figure 8:**
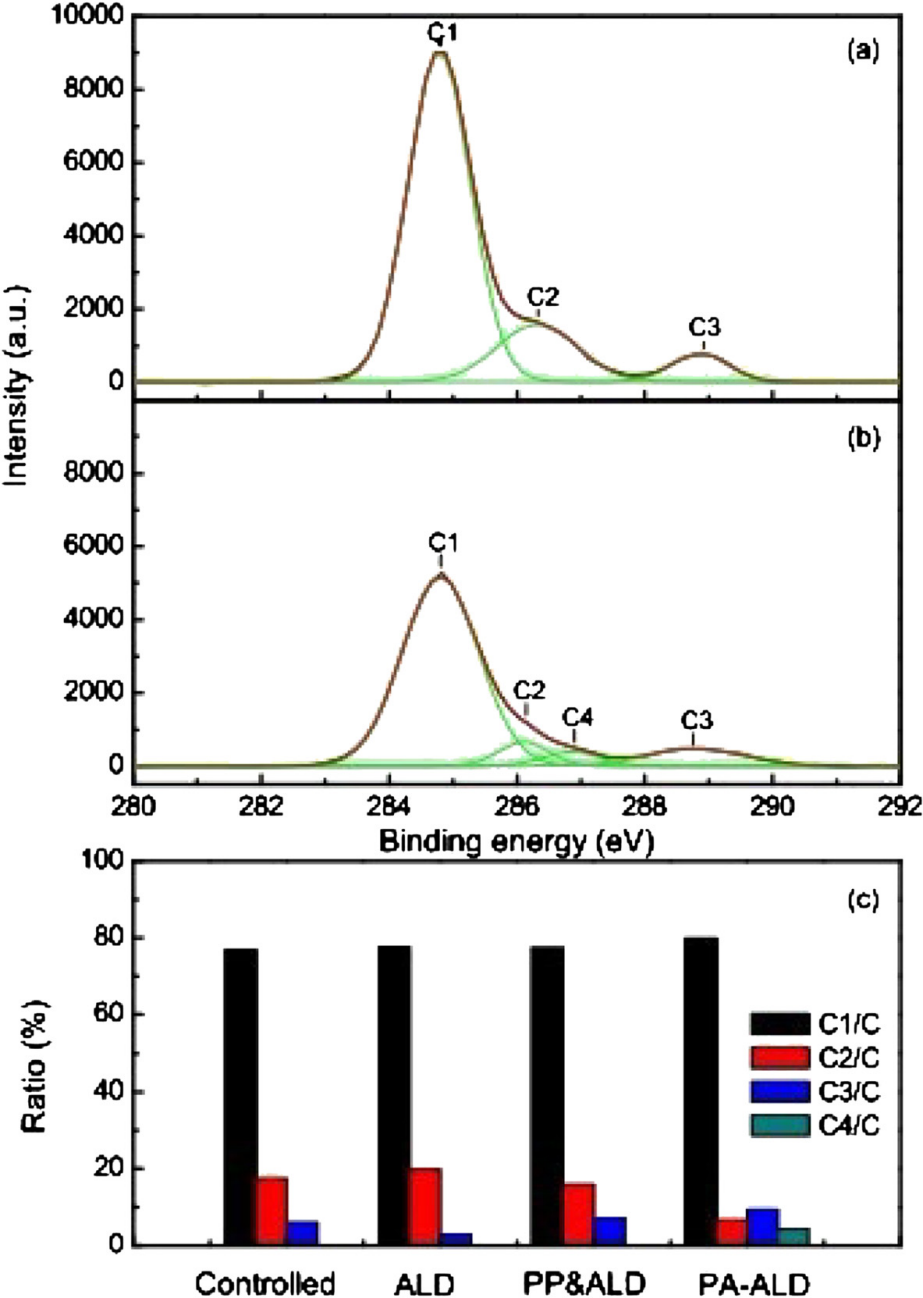
**XPS spectra of C 1*s *peaks.** With **(a)** uncoated PET, **(b)** the Al_2_O_3_-coated PET film by PA-ALD, and **(c)** relative elemental contents.

**Figure 9:**
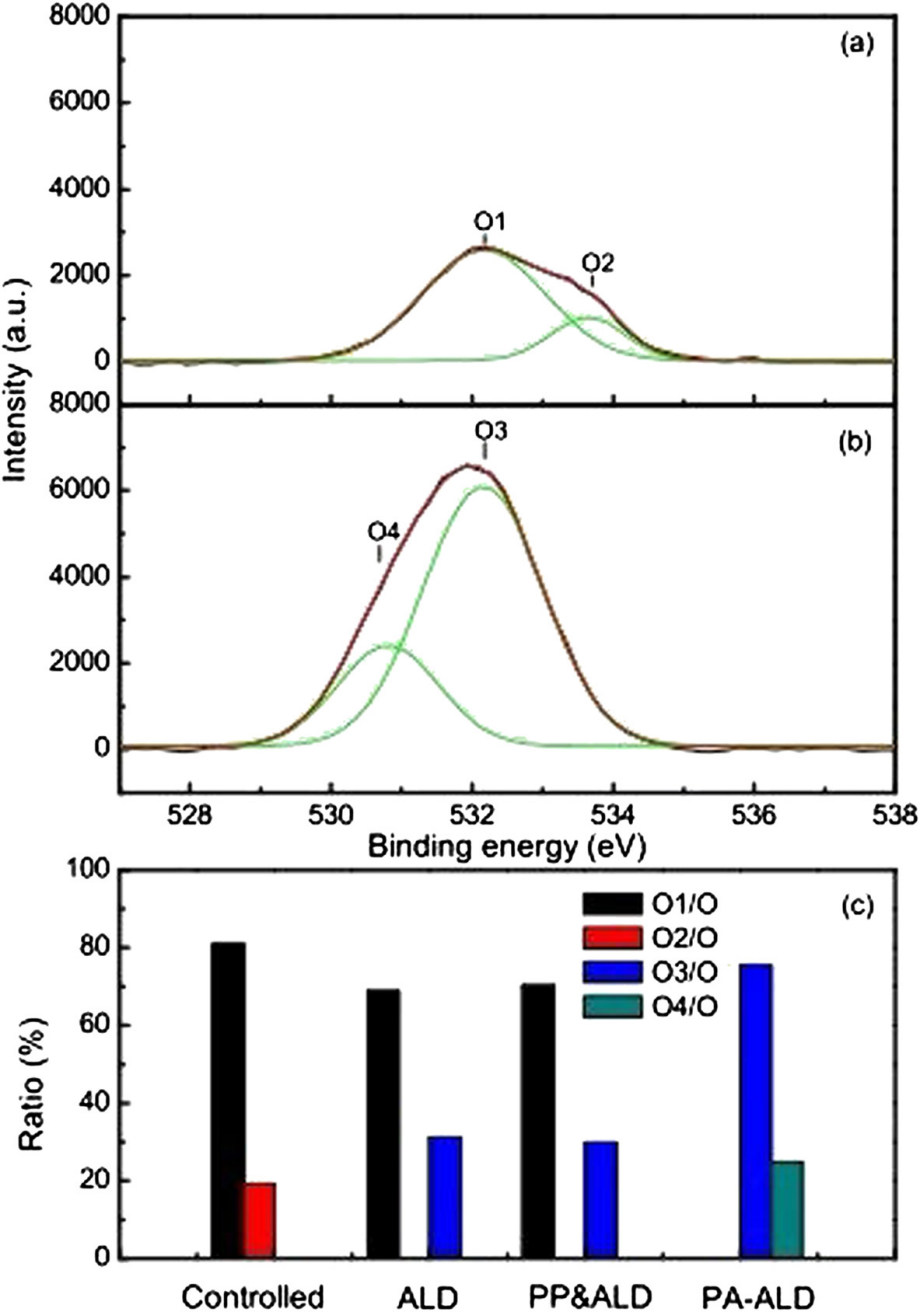
**XPS spectra of O 1*s *peaks.** With **(a)** uncoated PET, **(b)** the Al_2_O_3_-coated PET film by PA-ALD, and **(c)** relative elemental contents.

Surface modification in terms of wettability obtained by ALD with and without plasma assistance is dependent on the chemical composition of the deposited Al_2_O_3_ films, which is revealed by the XPS spectra of the uncoated and coated PET film, as shown in Figure 7. It shows the peaks at the binding energies of 284 and 531 eV, corresponding to the C 1*s* and the O 1*s*, respectively, with the uncoated PET film, as shown in Figure 7a. With the deposition of Al_2_O_3_ film by PA-ALD, another peak at the binding energy of 74 eV, corresponding to the Al 2*p*, is found in Figure 7b, and the relative content of O 1*s* is elevated, both of which are confirmed by the relative element contents shown in Figure 7c. The increment of O 1*s* content and the emergence of Al 2*p* are achieved for the Al_2_O_3_ film deposited by ALD, plasma pretreated ALD, and PA-ALD. Further investigation on the chemical structure of the uncoated and the coated PET surface are carried out by the high-resolution XPS analysis of C 1*s*, O 1*s*, and Al 2*p*. The concentration of each chemical component of C1*s* and O1*s* is examined by using Gaussian fit and shown in Figures 8 and 9.

Figure 8a gives the XPS spectra of C 1*s* with the uncoated PET, which show the peaks of C1, C2, and C3, in the range of 284.77 to 284.80 eV, 285.47 to 286.32 eV, and 288.84 to 289.05 eV, corresponding to the -C-C- (and C-H bonds), the -C-O (and/or -C-OH), and the O=C-O (and/or COOH), respectively, which are consistent with the published data on PET film [25-27]. In Figure 8b with the Al_2_O_3_-coated PET films by PA-ALD, the spectra show another peak of C4 at 286.86 eV, corresponding to the -C-OH, besides the peaks of C1, C2, and C3, which indicates that a new chemical state is formed on the Al_2_O_3_-coated PET by PA-ALD. As shown in Figure 8c, the appearance of C4 is followed by the reduction of C2 peak amplitude significantly, which indicates the presence of -C-OH on the PET surfaces [25,26]. The improvement on the formation of hydroxyl groups in PA-ALD is consistent with the FTIR results shown in Figure 6 that the highest amplitude of hydroxyl groups at the band of 3,429 cm^−1^ is also achieved by PA-ALD.

Figure 9a,b shows the O 1*s* peaks of uncoated PET and the Al_2_O_3_-coated PET film by PA-ALD. It shows that the spectrum of uncoated PET consists of O1 and O2 at the range of 531.43 to 532.16 eV and 533.64 eV, corresponding to the C=O and the C-O-, respectively [25]. On the other hand, the spectrum of Al_2_O_3_-coated PET film by PA-ALD consists of O3 and O4 at the range of 532.16 to 532.54 eV and 530.72 to 530.81 eV, corresponding to the Al_2_O_3_ (and Al-O-C) and the O in AlO of AlOOH, respectively [25,28]. It proposes the different deposition mechanism and dynamics during the ALD process. The detailed relative elemental contents of the uncoated PET and the Al_2_O_3_-coated PET films by ALD, plasma pretreated ALD, and PA-ALD are presented in Figure 9c. It shows that the Al_2_O_3_-coated PET films by ALD and plasma pretreated ALD consist of O1 and O3, which suggests that the element of C-O- is replaced by Al_2_O_3_ (and Al-O-C) during the ALD process. By introducing plasma in the ALD process, both the elements of C=O and C-O- are replaced by Al_2_O_3_ (and Al-O-C) and AlO in PA-ALD, which suggests the elimination of the CO-related elements and secures a normal growth of alumina oxide film on the PET film.

## Conclusions

The successful deposition of Al_2_O_3_ film on PET is achieved by ALD, plasma pretreated ALD, and PA-ALD, which is demonstrated by surface morphology and chemical composition of the deposited Al_2_O_3_ film. The introduction of plasma in the ALD process is found to be crucial for the initial growth of ALD deposition by forming the chemical functional groups, such as hydroxyl -OH group, which is also mostly responsible for the enhancement of surface wettability in terms of water contact angle. Another issue concerning energetic ion bombardment has to be taken into account with the application of plasma, which induces the cracks on the deposited films. The characterization of chemical composition shows the formation of AlO element and elimination of CO-related elements in the Al_2_O_3_ film deposited by PA-ALD with plasma in the ALD process, which is different from that by ALD and plasma pretreated ALD and proposes a normal growth of Al_2_O_3_ on the polymer of PET in PA-ALD.

## Competing interests

The authors declare that they have no competing interests.

## Authors’ contributions

RE participated in the design of the study, carried out the experiments, performed the analysis, and drafted the manuscript. XH participated in the experiment and prepared the devices for experiment. YG participated in revising the manuscript and carried out the XPS characterization. JZ participated in the design of the study and revised manuscript. JS participated in the design of the experiment, performed the analysis, and organized the final version of the paper. All authors read and approved the final manuscript.
